# Family environment variables and related health outcomes in children and adolescent and younger adults affected by cancer: a scoping review

**DOI:** 10.3389/fpsyg.2026.1843357

**Published:** 2026-07-13

**Authors:** Heeyeon Son, Patricia N. E. Roberson, Gianna Wood, Yuri Park, Kelsey West, Lauren Ghazal

**Affiliations:** 1 College of Nursing, University of Tennessee, Knoxville, TN, United States; 2 Vanderbilt University Medical Center, Nashville, TN, United States; 3 School of Nursing, University of Rochester, Rochester, NY, United States; 4 Wilmot Cancer Institute, Rochester, NY, United States

**Keywords:** adolescents and young adults, family environment, pediatric cancer, physical functioning, psychological outcomes, scoping review

## Abstract

Children, adolescents, and younger adults (AYAs, 0–24 years) affected by cancer often experience substantial physical and psychological symptom burden. The family environment may be an important yet underused and modifiable factor that supports physical functioning and psychological well-being. However, existing evidence remains fragmented and has not been comprehensively mapped. This scoping review aimed to identify: (1) the family environment characteristics examined in pediatric and AYA cancer research, and (2) the health outcomes associated with these factors. We searched PubMed, Web of Science, PsycINFO, and Scopus between January 6 and April 18, 2025, and identified 764 records. After full-text review, 24 studies met inclusion criteria. Most studies were cross-sectional (*n* = 19) and quantitative (*n* = 23), with one experimental study. Family environment variables were grouped into two categories: family process and relational factors, such as family functioning, and sociodemographic and contextual factors, such as socioeconomic status. Health outcomes were categorized as psychological or physical, although most studies focused on psychological outcomes. Across studies, more supportive family environments, including stronger family functioning, better socioeconomic conditions, and better parental mental well-being, were generally associated with improved psychological well-being and higher physical functioning. Most studies emphasized family process and relational factors, while relatively few examined sociodemographic and contextual factors. Similarly, psychological outcomes were assessed more often than physical functioning and health-related behaviors, such as smoking, substance use, or screen time. Interpretation is limited because most studies were cross-sectional, relied on self-report data, and were not guided by explicit theoretical frameworks. Still, the overall findings suggest that family-focused strategies may be promising for improving symptom management and well-being among children and AYAs affected by cancer.

## 1 Introduction

Despite advances in treatment for cancer, a cancer diagnosis remains most burdensome for children, adolescents and young adults (AYAs). Approximately 25,000–30,000 children and AYAs (ages 0–24 years) are diagnosed with cancer in the United States each year (Siegel et al., 2024; National Cancer Institute, 2025) and survival rates are a little over 86% (National Cancer Institute, 2025). The impact of cancer does not cease at treatment completion but continues throughout survivorship. During the survivorship, children and AYAs affected by cancer are continually challenged with symptoms attributed to intensive medical procedures endured during recovery, such as chemotherapy, or to the hardships of the malignancy itself (Torres et al., 2019). These treatment-related symptoms, including increased fatigue, pain, nausea, and sleep disturbances, impair both physical and psychological health, resulting in disrupted Health-related Quality of life (HRQoL) (Hockenberry et al., 2017). According to the National Childhood Cancer Survivor Study, long-term childhood cancer survivors are 80% more likely to report a clinically relevant impairment in mental health than their siblings. Physical impairments that affect endocrine, musculoskeletal, neurological, and cardiopulmonary systems are also common among those with childhood cancer diagnosis (Wilson et al., 2015). As a result, childhood cancer survivors are over five times as likely to report a declined HRQOL and twice as likely to report clinical levels of emotional distress (Hudson et al., 2003; Hudson et al., 2015). For this reason, research aimed to prevent and treat these long-term hardships have been identified as a national priority with the passing of the *Childhood Cancer Survivorship, Treatment, Access, and Research Act of 2018* (STAR Act, 2018). In 2020, the National Cancer Institute launched the Childhood Cancer Data Initiative (CCDI) to further understand cancer biology in improving preventative measures, treatment, HRQOL, and survivorship and is supported with more than 5 million dollars of funding annually (Sharpless, 2025).

Identifying modifiable factors influence physical and psychological health is essential to ultimately promote HRQoL among children and AYAs affected by cancer. Accordingly, researchers have identified many factors that have significant association with physical and psychological health outcomes: Fatigue during treatment, increased number of late effects, ethnic minority status, brain tumor diagnosis (Meeske et al., 2007), and other existing socioeconomic factors (Li et al., 2024; Oprandi et al., 2022). Unfortunately, modifying these factors is not straightforward in practice (Astorino et al., 2024). However, identifying modifiable moderators that mitigate their effects on physical and psychological health outcomes may be an important first step (Rosenberg and Salsman, 2024).

Specifically, family environment is of the most potent, yet underutilized modifiable factors to improve physical and psychological health outcomes and ultimately to promote HRQoL for those children and AYAs affected by cancer (Patterson et al., 2024). In health science research, family environment generally refers to the social climate and conditions within the family that shape health, behavior, and development (Bush et al., 2020; Wäsche et al., 2021). The specific examples include interpersonal relationships, communication, cohesion, conflict, support, organization, norms, and health-related routines or values within the household (Bush et al., 2020; Lanz and Maino, 2014; Wäsche et al., 2021). Numerous theories emphasize the important role of the family environment in influencing psychological outcomes, by allowing adolescents and children to better cope and adapt to life with cancer. For example, the Double ABCX-Model (McCubbin and Patterson, 1983) emphasizes the importance of how families perceive a cancer diagnosis as a crisis, as well as the resources available to them in responding to that crisis. The Family Adjustment and Adaptation Response Model (McCubbin and Patterson, 1983), an expansion of the Double ABCX-model, also explains how balance is maintained through the family’s available resources and coping mechanisms in navigating stressors, specifically when facing the challenges of adjustment, response to crisis, and ability to adapt. Furthermore, the Family Resilience Process Model explains family resilience in the face of maintaining functioning long-term across three dimensions of family belief systems, organizational patterns, and communication process (Walsh, 2003). Family Stress Model demonstrates how social barriers that disrupt family functioning influence child health outcomes (Masarik and Conger, 2017). Biobehavioral Family Model (BBFM) is relatively less applied in cancer context, but was used to explain how family dynamics influence individual health symptoms and severity, which is known as biobehavioral reactivity, or the pattern of psychological and emotional response to stress (Lim et al., 2022; Wood et al., 2008). Finally, the Bronfenbrenner’s (1979) ecological framework is one of the most well-known and widely applied frameworks. It emphasizes the role of multiple social systems and resources surrounding children and adolescents in shaping persons’ wellbeing, including family, school community, and broader sociocultural contexts. The framework is widely applied in various context, including pediatric cancer context, describing the family system as one of the most important variables in the lives of children with chronic illness (Kazak et al., 2003).

Currently, relatively more evidence exists supporting the influence of family functioning on psychological health outcomes—such as adaptability measures, and the occurrence of PTSD, depression, and anxiety (Robinson et al., 2007; Van Schoors et al., 2019). According to meta-analysis conducted by Van Schoors et al. (2017), family functioning is one of the most widely examined constructs in family studies, encompassing specific domains, such as cohesion and communication, and were identified as consistent influences on adjustment among children with cancer. Despite this, children’s physical health outcomes have not been systematically investigated within this framework, leaving gaps in our understanding on which specific aspects of family environment contributing to improved health outcomes. Despite the importance of family environment, translating the knowledge into the development of family-based interventions can be challenging, given that there is no consensus on which factors at family environment need to be targeted. Therefore, studies that synthesize existing empirical knowledge are urgently needed to examine the development of effective family-based interventions in improving HRQoL among children and AYAs affected by cancer. Accordingly, we conducted a scoping review to examine: (1) family environment variables associated with physical functioning and psychological well-being in children, adolescents, and young adults (AYAs; ages 0–24) affected by cancer, and (2) the related health outcomes. Finally, the family environment variables and related health outcomes are depicted according to the ecological framework.

## 2 Methods

### 2.1 Data sources and search strategies

A health sciences librarian with expertise in systematic review methodologies assisted with the selection of databases and the development of search terms. We completed a systematic search from January 6, 2025, to April 18, 2025, for relevant articles indexed in PubMed, PsycINFO, Cumulative index of Nursing and Allied Health Literature (CINAHL), and Scopus. The combination of keywords and controlled vocabulary terms related cancer, family environment, and adolescent/young adults were used to combine search concepts. Within each concept, synonyms were combined with OR, and major concepts were combined with AND. The example of mesh terms and applied Boolean operators include: child* OR adolescen* OR teen* OR baby OR babies OR infant* OR youth OR paediatric OR pediatric, (cancer* OR neoplasm* OR oncolog*) AND survivor*, (famil* OR home OR hous* OR dwelling OR residence*) AND (environment*OR surrounding* OR setting*), and (physical OR psychological OR mental) And (outcome*OR effect or impact or influence). Filters were applied based on inclusion criteria and include English language, peer-reviewed articles, human subjects, and publication year from 2015 to 2025. Search strategies were adapted for each database based on available subject headings and indexing terms. We also reviewed references of review articles and screened articles for additional literature.

### 2.2 Inclusion and exclusion criteria

Inclusion criteria were: (1) full-text article in a peer-reviewed journal, (2) published in the English language, (3) published from January 2015 through January 2025, (4) presented original research which examined influence of family environment on physical or psychological health outcomes among children and AYAs affected by cancer (0–24 years) in order to capture the age period during which individuals are most plausibly living with and under the influence of caregivers or families that is supported by several strands of evidence (Loo, 2024; Centers for Medicare and Medicaid Services, 2025; U.S. Department of Labor, 2025), and (5) included at least one family environment variable and outcomes concerning physical or psychological health. To explore this range of research, we included both qualitative and quantitative research studies. Experimental/interventional studies were also included if they had correlational results between family environment factors and physical or psychological health outcomes. Studies other than full research articles (e.g., commentaries, research protocols, conference abstracts, dissertations), studies published in language other than English, and studies addressing the association between family environment factors and health outcomes in siblings or parents of cancer patients were excluded.

### 2.3 Procedure

**Figure 1:**
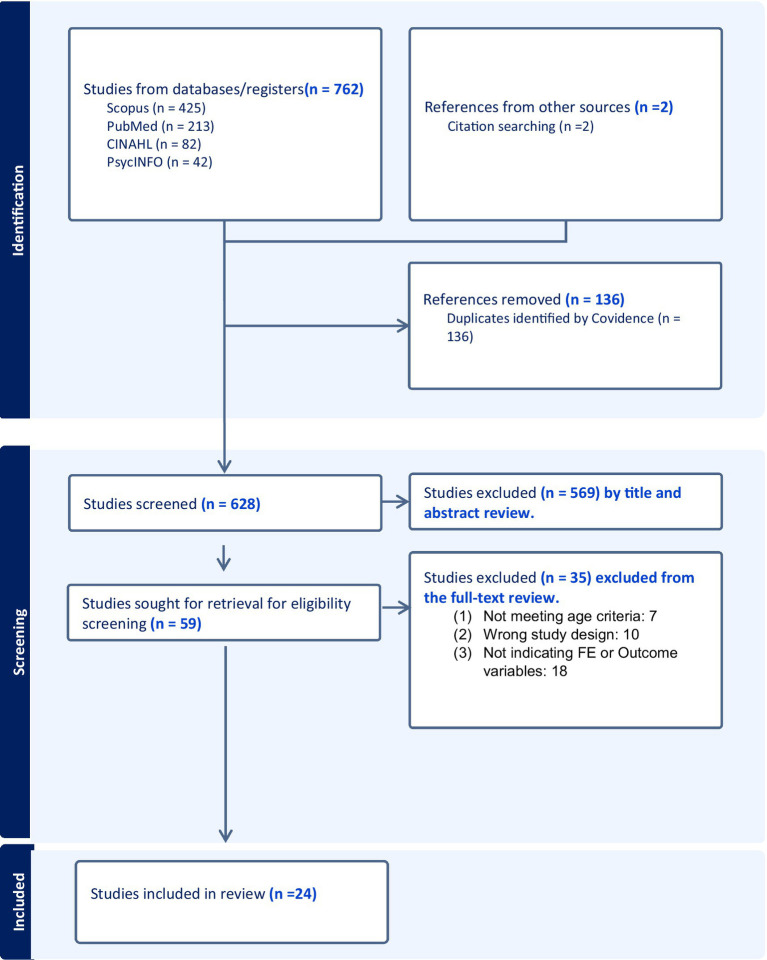
PRIMSA 2020 flow diagram of the study selection process, adapted from Tricco et al. (2018).

Employing PRISMA-ScR scoping review methods (Tricco et al., 2018), our initial search yielded 764 articles, including two articles from manual search. We used Covidence software to remove 136 duplicates and then screened 628 abstracts. Two team members (GW and YP) independently screened each abstract, and conflicts were reconciled through consensus with one author (HYS), being the final arbiter. We then screened 59 full-text articles following the inclusion criteria. The PRISMA flow diagram for the screening process is in Figure 1.

### 2.4 Extraction and charting of results

Each reviewer (GW, YP, and HYS) read the full-text articles and had discussions with each other to determine whether the articles satisfied the inclusion and exclusion criteria, as well as the strengths and weaknesses of each study to be included. The three reviewers independently completed an investigator-developed table to extract key study information, regarding the study design, methods, participant characteristics, study measures, family environment variables, guiding theoretical framework, and relevant health outcomes. This information was independently verified by two other researchers (PR and LG), and articles were organized by study method. The three authors independently developed themes related to family environment variables and the relevant health outcomes through discussion.

## 3 Results

### 3.1 Overview of included studies

The initial search included all studies published between January 2015 and January 2025. As outlined in Figure 1, a total of 762 articles were initially retrieved through the database search. Two additional articles were identified through cross-references and hand searching, yielding a total of 764. Of these, 59 articles were retrieved for full-text review, and 24 studies were retained for the final analysis. A summary of the studies included in this review is presented in Supplementary Table S1. Evidence from the 24 included articles organized by study design (cross-sectional/longitudinal, quantitative, qualitative or mixed design study) with study and participant details, measures used, family environment variables (independent variables), physical or psychological health outcomes reported, and the theoretical framework used in the study. The key study findings about the family environment variables, relevant health outcomes, and the association between them are organized according to Bronfenbrenner’s ecological model (Bronfenbrenner, 1979, Figure 2). The summary of the variables is presented in Figure 3.

**Figure 2:**
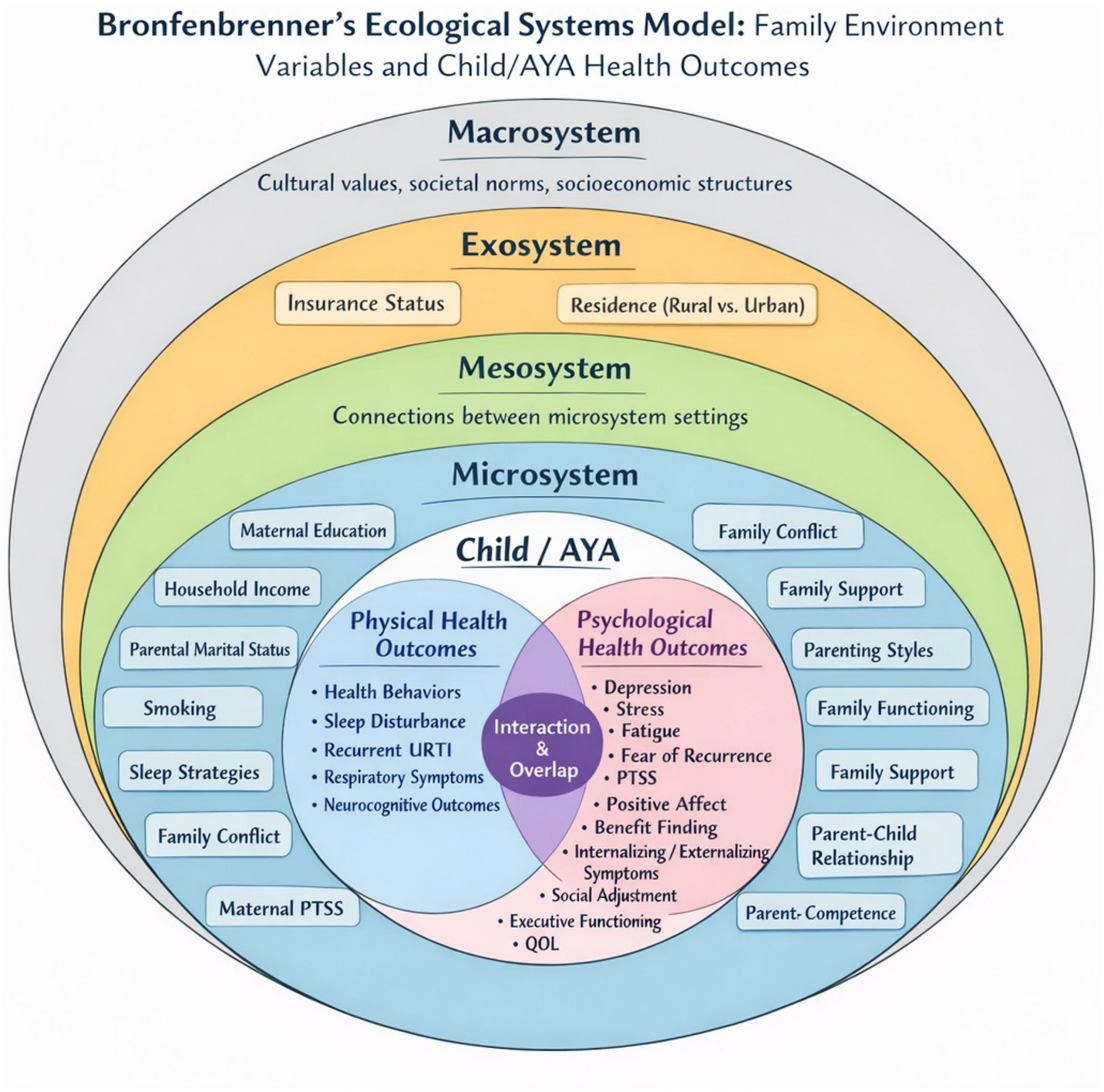
Conceptual Framework of Family Environment Variables and Child/AYA Health Outcomes, adapted from Bronfenbrenner’s Ecological System Theory (Bronfenbrenner, 1979).

**Figure 3:**
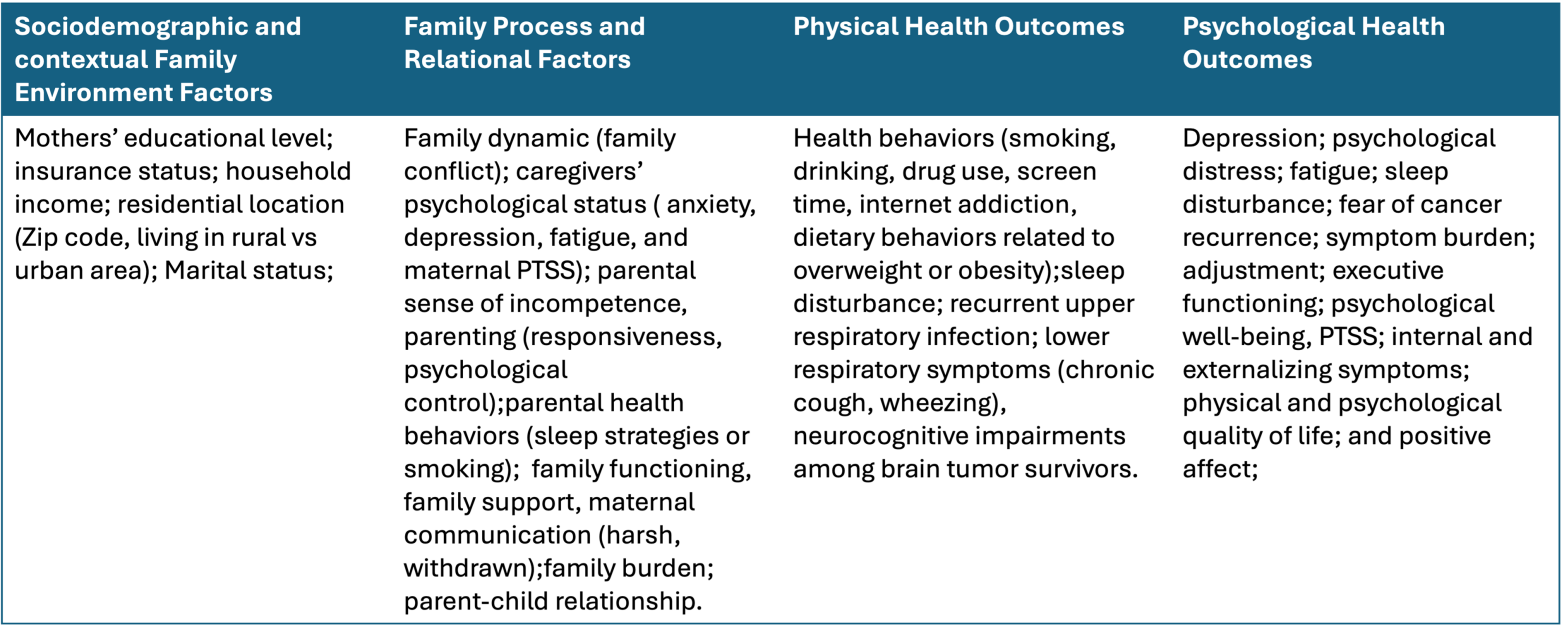
Summary of family environment variables and relevant health outcomes.

#### 3.1.1 Study characteristics

Majority of studies included in this review employed cross-sectional designs (Al Ghriwati et al., 2021a,b; Cousino et al., 2017; Delehaye et al., 2024; Erickson et al., 2022; Gutiérrez-Colina et al., 2017; Hocking et al., 2017; Huang et al., 2018; Kim and Yoo, 2015; Moscato et al., 2025; Racine et al., 2018; Sim et al., 2024; Tillery et al., 2020; Winning et al., 2022; Žarković et al., 2024; Zheng et al., 2022). Six studies out of 24 employed longitudinal design studies (Kunin-Batson et al., 2016; Murphy et al., 2016; Patterson et al., 2024; Quast et al., 2018; Tillery et al., 2022; Van Laere et al., 2023). There was one mixed-design study (Kim et al., 2020) and one randomized control trial (Bidstrup et al., 2023). The majority of studies were conducted in the United States (Al Ghriwati et al., 2021a,b; Cousino et al., 2017; Erickson et al., 2022; Gutiérrez-Colina et al., 2017; Hocking et al., 2017; Huang et al., 2018; Kim et al., 2020; Kunin-Batson et al., 2016; Moscato et al., 2025; Patterson et al., 2024; Quast et al., 2018; Sim et al., 2024; Tillery et al., 2020, 2022; Winning et al., 2022), and Denmark (Bidstrup et al., 2023), Canada (Racine et al., 2018), France (Delehaye et al., 2024), South Korea (Kim and Yoo, 2015), Switzerland (Žarković et al., 2024), Belgaum (Van Laere et al., 2023), and China (Zheng et al., 2022).

#### 3.1.2 Study sample characteristics

The age of children and AYA participants ranged from 2 to 24 years old. Four studies targeted younger children (Bidstrup et al., 2023; Kim et al., 2020; Kunin-Batson et al., 2016; Tillery et al., 2020) and five studies included adolescents and young adults (Cousino et al., 2017; Gutiérrez-Colina et al., 2017; Tillery et al., 2022; Van Laere et al., 2023) and 15 studies included a mix (Al Ghriwati et al., 2021a,b; Delehaye et al., 2024; Erickson et al., 2022; Hocking et al., 2017; Huang et al., 2018; Kim and Yoo, 2015; Moscato et al., 2025; Patterson et al., 2024; Quast et al., 2018; Racine et al., 2018; Sim et al., 2024; Winning et al., 2022; Žarković et al., 2024; Zheng et al., 2022). Majority of studies, 14 out of 24 collected self-reports from the children/AYAs-caregiver dyads; however, eight studies included either caregiver’s self-report only (Hocking et al., 2017; Kim et al., 2020; Žarković et al., 2024) or child/AYAs’ responses only (Delehaye et al., 2024; Erickson et al., 2022; Gutiérrez-Colina et al., 2017; Huang et al., 2018; Zheng et al., 2022). Bidstrup et al. (2023) conducted an intervention study with whole family members of children affected by cancer. Majority of the study participants were cancer survivors who had just completed cancer-treatment (Al Ghriwati et al., 2021a; Al Ghriwati et al., 2021b; Cousino et al., 2017; Delehaye et al., 2024; Erickson et al., 2022; Gutiérrez-Colina et al., 2017; Hocking et al., 2017; Huang et al., 2018; Kim and Yoo, 2015; Kunin-Batson et al., 2016; Moscato et al., 2025; Patterson et al., 2024; Racine et al., 2018; Sim et al., 2024; Tillery et al., 2020; Tillery et al., 2022; Van Laere et al., 2023; Winning et al., 2022; Žarković et al., 2024; Zheng et al., 2022). Only few studies included in this study focused on participants under the active treatment (Kim et al., 2020; Murphy et al., 2016).

### 3.2 Family environment variables in obtained studies

#### 3.2.1 Sociodemographic and contextual factors

Across literature, there were a verity of family environment variables and were categorized into sociodemographic and contextual factors. Within the ecological framework, the family sociodemographic and contextual factors were categorized into either the microsystem or exosystem (Figure 3). Across the included studies, these variables include mothers’ educational level (Huang et al., 2018; Quast et al., 2018; Zheng et al., 2022), insurance status (Sim et al., 2024), household income (Kunin-Batson et al., 2016; Zheng et al., 2022), and residential location (Sim et al., 2024; Zheng et al., 2022), such as rural vs. urban living, or poverty area, parents’ marital status (Sim et al., 2024). These variables do not have direct impacts on child’ or AYAs’ health outcomes but impact them indirectly affecting parental capacity to provide resources and interact with child and AYAs, in turn directly affect them. Among those variables, mothers’ education level, insurance status, household income, and residential location, and parental marital status are variables that most cited as social factors influencing health (Kiernan and Huerta, 2008; Wilkinson and Marmot, 2003).

#### 3.2.2 Family process and relational factors

The majority of identified family environment variables were categorized into family process and relational factors. These factors also can be described as microsystem according to ecological system, which has direct impact on persons’ health outcomes (Bronfenbrenner, 1979; Figure 2). Those included family conflicts (Sim et al., 2024) caregivers’ emotional and mental health conditions, including anxiety (Sim et al., 2024) depression (Gutiérrez-Colina et al., 2017), and fatigue (Sim et al., 2024). Parenting patterns were also frequently assessed and this includes parental sense of incompetence, parents’ responsiveness (Van Laere et al., 2023), along with parents’ psychological control and overprotection (Van Laere et al., 2023; Winning et al., 2022). Family support, maternal post-traumatic stress syndrome (PTSS), and maternal communication were also explored (Murphy et al., 2016). Other family environment variables that belong to family process and relational factors include family functioning, parental sleep strategies (Kim et al., 2020), which are behaviors that parents use to put their children to sleep, and parental smoking (Žarković et al., 2024). Of all family environment variables, family functioning was the most frequently examined in relation to psychological well-being in children and AYAs affected by cancer (Patterson et al., 2024; Tillery et al., 2022; Tillery et al., 2020).

### 3.3 Physical and/or psychological health outcomes in obtained studies

A relatively small number of studies examined physical health outcomes in relation to family environment among children and AYAs affected by cancer. For example, one study examined childhood cancer survivors’ health behaviors, such as smoking, drinking, drug use, screen time, internet addiction, and dietary behaviors linked to overweight or obesity (Zheng et al., 2022). Another study examined sleep disturbances among childhood cancer survivors as influenced by parental sleep strategies (Kim et al., 2020), and the other study examined the incidence of upper respiratory tract infection as an outcome of parental smoking (Žarković et al., 2024). Finally, another study examined neurocognition outcomes (e.g., executive functions) among brain tumor survivors (Hocking et al., 2017). Despite these few, there is a clear lack of exploration in physical symptom correlation with family function.

A comparatively larger number of outcomes related to psychological health outcomes were examined across the literature, most of which focused on negative indicators such as depressive symptoms or depression (Gutiérrez-Colina et al., 2017; Kunin-Batson et al., 2016; Sim et al., 2024; Van Laere et al., 2023), fear of cancer recurrence (Van Laere et al., 2023), PTSS (Murphy et al., 2016; Tillery et al., 2020), internal and externalizing symptoms (Al Ghriwati et al., 2021a; Cousino et al., 2017; Winning et al., 2022), symptom burden (Huang et al., 2018), and psychological distress (Bidstrup et al., 2023). The positive outcomes explored included: benefit finding (Van Laere et al., 2023), survivors’ social adjustment (Hocking et al., 2017), and survivors’ functioning (Hocking et al., 2017; Moscato et al., 2025). Again, across the literature, the most widely explored outcomes were depression/depressive symptoms, rather than physical ones.

### 3.4 The influences of family environment on health outcomes

Across the studies we examined, the family environment had a substantial and consistent impact on the physical and psychological health outcomes among children and AYAs affected by cancer. The overall correlation between family environment variables and health outcomes was positive (i.e., worse family functioning is associated with worse health outcomes).

#### 3.4.1 The influence of families’ sociodemographic and contextual factors on health outcomes

In most studies, social economic status (SES) of families, as described sociodemographic and contextual factors in this study, was not the main interest of study; however, the influence of SES on either physical functioning or psychological well-being has been explored in several studies as it accounts for the economic and structural aspects of the family. Delehaye et al. (2024) examined the association between SES and psychosocial difficulties in childhood cancer survivors and found that greater social deprivation of their families was significantly associated with children with cancers’ learning difficulties at school but not with psychological difficulties. Among the various SES variables, maternal education level was most frequently identified significant predictor of outcomes. In Quast et al. (2018), researchers found that maternal education level was related to mother-rated survivors’ HRQOL at nine-months follow-up. In another study, lower maternal education was related to more child symptom burden and poorer HROQL (Erickson et al., 2022). However, Patterson et al. (2024) did not find a link between SES and hair cortisol concentration in pediatric cancer survivors. Finally, Zheng et al. (2022) found that home environment factors, such as residential location, parents’ education level, and household income, along with family support, directly affected the CCS’s screen time, internet addiction, and dietary behaviors among Chinese childhood cancer survivors (CCS).

#### 3.4.2 Parents’ psychological status influences physical and psychological health outcomes

Parental psychological status, particularly maternal psychological status, emerged as one of the major family process and relational factors within the ecological framework, contributing to health outcomes, most notably psychological well-being, across the included studies. In Sim et al. (2024), caregiver’s high anxiety was significantly associated with survivors’ depression, increased stress, fatigue, sleep issues, and reduced positive affect. Al Ghriwati et al. (2021b) investigated associations among cancer- and treatment-related variables, family factors—including caregiver distress, caregiver HRQOL, and general family functioning—and patients’ HRQL, drawing on reports from both caregiver and children and adolescents with cancer. They found that lower caregiver distress predicted better survivors’ psychological and physical HRQOL. Huang et al. (2018) similarly found that higher parental emotional distress was associated with poor health status and HRQOL among acute lymphocytic leukemia (ALL) survivors. Parental stress was additionally associated with family strain due to worsening child symptom burden and poor child HRQOL. Racine et al. (2018) found that parental psychological distress and psychosocial family risk were negatively associated with both child-reported and parent-reported child HRQOL. The parent-reported psychosocial family risk also moderated the association between parent psychological distress and parent-reported child HRQL. The only randomized control trial by Bidstrup et al. (2023) also supported the influence of the parents’ psychological status on the child’s outcomes. In this study, Bidstrup tested the effect of Family-Oriented Support (FAMOS) family therapy program on reducing parent-reported medical traumatic stress among a pediatric cancer survivors ages 2–5 years old. The FAMOS is family-based psycho-therapeutic family intervention, which was designed to alleviate psychological symptoms in the whole family after the end of childhood cancer treatment, through cognitive behavioral, problem-solving, and goal setting interventions that aim to improve individuals’ functioning (Salem et al., 2021). Bidstrup and colleauges reported that the FAMOS was effective in reducing trauma-related behaviors in children by reducing depressive symptoms in mothers, but reducing depressive symptoms in fathers had little effect.

#### 3.4.3 Healthy family functioning influences both physical and psychological health outcomes

Family functioning was among the most widely studied family environment variables. Family functioning was studied in a total of eight studies. Positive family functioning is generally characterized by effective problem-solving, clear and direct communication, appropriate emotional responsiveness, empathy, and flexible, reasonable handling of daily challenges. Hocking et al. (2017) found that overall positive family functioning predicted improved executive functioning in pediatric brain tumor survivors. In Hocking’s study, parents reported that their perception of child’s behavior and development influenced how they supported their children’s social functioning during survivorship. Moscato et al. (2025) found that family functioning was associated with both physical and psychological HRQoL for children receiving moderate intensity cancer treatments. Gutiérrez-Colina et al. (2017) found that poor family functioning perceived by caregivers and adolescent and younger adult cancer survivors correlated with higher depressive symptoms among AYA survivors. Additionally, AYAs’ perception of family functioning seemed to predict caregivers’ perception of depressive symptoms as well.

Kunin-Batson et al. (2016) also focused on the role of family functioning and they found that poor family functioning, measured using McMaster Family Assessment Device, was associated with anxiety and depression among childhood acute lymphoblastic leukemia (ALL) survivors. The poor family functioning was characterized by difficulties in problem-solving, communication, role-assignment, emotional responsiveness, involvement, and behavior control. In Quast et al. (2018), worse family functioning predicted worse survivor-rated HRQOL at a nine-month follow-up. Erickson et al. (2022) investigated the association between family functioning over time and hair cortisol concentration among pediatric cancer survivors. Parents’ hair cortisol concentration and parent-reported family functioning—particularly the “independence” dimension (i.e., the extent to which family members are assertive, self-sufficient and make their own decisions)—significantly associated with pediatric cancer survivors’ hair cortisol concentration. While psychological health outcomes were the most frequently examined outcomes in relation to family functioning, Al Ghriwati et al. (2021b) examined both physical and psychological health outcomes by evaluating the associations among cancer- and treatment-related variables, family factors, survivors reported HRQoL, and neurocognitive difficulties. They found that family functioning was a significant predictor of survivors’ better physical and psychological health outcomes and HRQoL and was associated with fewer neurocognitive difficulties.

#### 3.4.4 How parenting shapes both physical and psychological health

Relatively little research has examined how parenting affects physical or psychological health outcomes. Van Laere et al. (2023) found that survivors’ reports of maternal and parental responsiveness positively predicted survivors’ benefit findings and negatively predicted survivors’ depressive symptoms. On the other hand, maternal reports of mothers’ responsiveness and overprotection positively predicted survivors’ fear of cancer recurrence and depressive symptoms. Winning et al. (2022) found the impacts of parenting on CNS brain tumor survivors’ adjustment (social competence, internalizing/externalizing problems, and academic competence). The researchers found that high levels of maternal behavioral control buffered the negative impact of CNS-directed treatment on children’s social competence. Maternal warmth had a contrasting effect, with worse academic competence being associated with CNS-directed treatment when mother showed a higher level of warmth. Huang et al. (2018) also found that excessive parental protective behaviors were associated with poorer survivors’ HRQoL and health status. For example, more parental protective behaviors were related to greater symptom severity (i.e., perceived abnormal physical, emotional, and cognitive symptoms) among childhood cancer survivors via indirectly through increased family strain. Finally, Kim and Yoo (2015) found that when parents experienced a greater difficulty in managing their child’s condition or reported less satisfaction in their ability to collaborate, the child was more likely to show evidence of psychosocial problems.

#### 3.4.5 The role of parent–child relationship in shaping physical and psychological outcomes

Three of the 24 articles included in this study examined the influence of parent–child relationships or overall family relationships on physical or psychological health outcomes. Al Ghriwati et al. (2021a) identified family relationship patterns in youth who recently completed cancer treatment and determined the character of the relationship between these patterns and children’s adjustment (internalizing, externalizing symptoms, and children’s social and overall functioning). In this study, childhood cancer survivors (CCS) from families with high levels of discord reported poorer adjustment, demonstrated through externalization of problems. Also, CCS who had low relational closeness and a lack of harmony and agreement or trust with other family members (such as siblings or parents) experienced difficulties in establishing peer relationships. Tillery et al. (2022) examined the longitudinal links between family environment variables and AYA cancer survivors’ substance abuse behaviors: problematic caregiver-AYA survivor relationships increased the likelihood of polysubstance use (PTSS) among AYA cancer survivors. Tillery et al. (2022) also explored the impacts of parent–child relationships on children’s psychological functioning, indicating that strained parent–child relationships were associated with elevated levels of PTSS, internalization of symptoms, and poor social functioning in children with cancer. Finally, Huang et al. (2018) found that lower family cohesion predicted poorer survivors’ HRQoL and poor health status among ALL survivors.

#### 3.4.6 Parent–child communication shapes the long-term health outcomes

Parent–child/adolescent communication is one of the important functions of the family reflecting the quality of parent–child relationships. Despite the importance of parent–child/adolescent communication (Branje et al., 2003), only two studies examined the influence of parent–child communication and relevant health outcomes. Murphy et al. (2016) found a significant longitudinal association between maternal PTSS and adolescent’s PTSS, through negative maternal communication. For example, greater maternal PTSS at study entrance often predicted harsher maternal communication at T2 (3 months). In addition, greater baseline maternal PTSS predicted adolescents’ elevated PTSS level at T3 (1 year), thought to be influenced by the fewer validations (communication) at T2 (3 months). Patterson et al. (2024) examined associations between early sociodemographic, family stress, and general mother-adolescent communication on the HRQOL of survivors at 5 years post-diagnosis. They found that adolescent stress, mother’s stress, and mother-adolescent communication significantly predicted mother-reported survivors’ HRQOL; however, only adolescent stress and mother-adolescent communication predicted adolescent self-report of stress.

#### 3.4.7 Additional relevant findings

Two studies examined associations among caregivers’ sleep disturbances, parental sleep-related accommodation behaviors, and children’s physical activity and sleep quality. Sim et al. examined the influence of diverse family- and community-level factors affecting patient reported health outcomes. Particularly, family-level factors included family dynamics, such as family conflicts, and caregivers’ self-report outcomes using PROMIS (Sim et al., 2024). Caregiver’s sleep disturbances were also significantly associated with lower physical activity in survivors and family conflicts were associated with survivors’ poor sleep quality. Kim et al. (2020) found that parental accommodation behaviors in sleeping, such as co-sleeping or comforting activities and parents’ psychological status (anxiety) were associated with children’s sleep disturbance. Finally, Cousino et al. (2017) demonstrated that the illness-specific family burden served as a mediator between cancer diagnosis-related late effects and survivors’ emotional and behavioral outcomes, thereby contributing to an elevated risk of parent-reported internalizing symptoms.

### 3.5 Theoretical frameworks explored in obtained studies

Based on the literature included in this study, we found that only six studies included theoretical frameworks (Al Ghriwati et al., 2021b; Cousino et al., 2017; Hocking et al., 2017; Kim and Yoo, 2015; Van Laere et al., 2023; Zheng et al., 2022). Al Ghriwati et al. (2021b) employed the Risk-and-Resilience-System to explain the association between family factors (caregiver distress, caregiver HRQOL, general family functioning) and patients’ HRQOL, using both caregiver- and self-report within 6 months after competing cancer treatment. Cousino et al. (2017) drew on the Transitional Stress and Coping Model (Thompson and Gustafson, 1996) to explain how parenting stress may contribute to poor behavioral functioning in children with cancer. Hocking et al. (2017) employed the Model of Social Competency to explore caregivers’ perspectives on survivor social competence and important risk/resilience factors by collecting both qualitative and quantitative data (Yeates et al., 2007). In Kim and Yoo (2015)‘s study, the authors examined the psychosocial problems in childhood cancer survivors in Korea and their connection to family management styles, using the Family Management Style Framework (FMSF) to categorize styles by type. Van Laere et al. (2023) employed Belsky’s Process Model of Parenting to examine the impacts of patterns of behavior and capability in parenting on childhood cancer survivors’ psychological functioning. Finally, Zheng et al. (2022) examined multiple health behaviors among Chinese childhood cancer survivors and explored the influencing factors at individual, interpersonal, and home environment levels under the guidance of socio-ecological framework (Bronfenbrenner and Morris, 2007). All findings from this study are presented in the synthesis model depicting the influence of family environment on health outcomes (Figure 2).

## 4 Discussion

This review aims to identify family environment variables that contribute to better physical and psychological health outcomes among children and adolescents and young adults affected by cancer. Additionally, the present scoping review highlights dominant areas of focus and identifies gaps within existing literature.

### 4.1 Family environment variables explored

First, the majority of family environment variables explored in the childhood cancer context were rooted from psychosocial aspects and described as family process and relational factors, according to ecological framework. The most widely examined family environment variable was family functioning, which has shown significant contributions to patient outcomes. Relatively few sociodemographic and contextual family environment variables were identified, including socioeconomic status (SES) variables and other social factors that influence individual health. According to the World Health Organization (2025), health is shaped by socioeconomic and environmental factors, including the conditions in which people are born, grow, live, work, and age. These conditions are further influenced by broader factors and systems, including socioeconomic status, education, physical environment, and social support. Family environment as one of forms of social support (Lewandowski et al., 2010), it is impossible to separate those family environment variables entirely from the socioeconomic and environmental factors. Accordingly, these family’s sociodemographic and contextual factors were classified as either micro or exosystem in this review guided by Bronfenbrenner’s ecological framework. However, there are diverse viewpoints regarding inclusion of SES as the family environment variable. For example, psychology, family systems research, and biopsychosocial research sees the family environment as a multi-dimensional, social and emotional climate inside the home (Kazak, 2006; Repetti et al., 2011; Rutter, 2006; Taylor et al., 1997). Thus, the family environment scale (FES) measures cohesion, expressiveness, conflicts, and independence to measure family environment (Moos, 1974). According to this perspective, social economic status of family members, such as parents’ income and mothers’ educational level, are not regarded as family environment variables. On the other hand, the Family Stress Model consistently shows that lower income or unstable employment undermines couple and parent–child relationships (Masarik and Conger, 2017). Based on our review and consistent with the general definition of family environment (Bush et al., 2020; Wäsche et al., 2021), we argue that a broader and more inclusive definition of family environment is needed. This approach may better guide future research by expanding attention beyond interpersonal factors, such as relationship quality, cohesion, and family members’ psychological status.

### 4.2 Health outcomes explored

Likewise, a relatively greater number of psychosocial outcomes were explored in terms of patient outcomes, compared to the physical health outcomes. The most widely explored psychological outcomes are depression/anxiety, meaning a rather great number of studies also focused on the influence of negative family environments on patient psychological health outcomes. Overall, the evidence linking family environment to physical health outcomes among children and adolescents with cancer was limited. Only four out of 24 included studies examined physical health outcomes or health-related behaviors that may influence physical outcomes. This contrasts with findings from research on other non-cancerous pediatric chronic illnesses, where family environment—particularly family functioning—has been consistently associated with physical health outcomes. For example, studies of children and adolescents with chronic pain have demonstrated that supportive family functioning is related to lower pain intensity and improved physical functioning (Lewandowski et al., 2010). The lack of similar evidence in pediatric oncology highlights an important gap and underscores the need for further investigation into how family environments may shape physical health trajectories in children and adolescents with cancer specifically. In terms of characteristics of health outcomes, only three studies explored the benefits of a supportive family environment, such as benefit finding, survivors’ social adjustment, and survivors’ exclusive functioning (Hocking et al., 2017; Moscato et al., 2025; Van Laere et al., 2023). Since around 2001, health research has shifted toward not just disease prevention and recovery, but also promoting positive health, well-being, and optimal human functioning. The positive health approach also highlights attention to how and why individuals thrive and flourish (Ryff and Singer, 2003). Therefore, considering the pivotal role families playing shaping individuals’ health and health-behaviors (Weiss-Laxer et al., 2020), it is essential that future research focuses on the contributions of families to positive health outcomes.

### 4.3 Relationship between family environment variables and health outcomes

It was evident that the family environment contributed to health outcomes among children and adolescents and younger adults in the cancer context; however, the ability to support the causal relationships between family environment variables and health outcomes was limited because the majority of studies included in this review employ cross-sectional designs.

#### 4.3.1 The influence of social economic status of family or individual members

One of the most frequently explored family environment variables was demographic characteristics, such as family socioeconomic status (SES) and family member characteristics. Despite this, the influence of SES on health outcome was consistently supported across the included literatures. For example, two out of four studies did not find a link between poor SES and health outcomes. This finding is inconsistent with previous findings, which have demonstrated influence of SES in shaping health outcomes among children with chronic illnesses. Previously, researchers found a significant link between social factors, such as age, ethnicity, social isolation, and access to care and prevalence and severity of specific cancer-related symptoms among children, adolescents and young adults with cancer (Asare et al., 2017; Sharara et al., 2024; Yim et al., 2021).

#### 4.3.2 The influence of psychological status of parents

Historically, parenting and parent–child relationships have been recognized as critical factors shaping the well-being of children and adolescents (Chen et al., 2017). In this review, we found evidence supporting a relationship between parental psychological status and patient outcomes. In obtained studies, healthier psychological functioning in parents was linked to improved patient outcomes. However, a key limitation of the existing literature is that most studies have focused of parental psychological status on psychological well-being of children and AYAs, while relatively few have examined physical health outcomes. When physical health outcomes have been addressed, they have often been included as components of health-related quality of life, such as pain and nausea (Al Ghriwati et al., 2021b; Huang et al., 2018; Racine et al., 2018). Additionally, few studies have examined the mechanisms underlying the association between parental psychological status and physical outcomes, highlighting the need for future research to further investigate this relationship.

#### 4.3.3 The influence of overall family functioning

Family functioning was one of the family environment variables that was most widely studied and a broad construct, encompassing several specific family domains. While family functioning has no universally accepted definition, in general, family functioning encompasses the social and structural characteristics of the family environment, including patterns of interaction and relationships among family members (Epstein et al., 1978; Epstein et al., 1983). It is commonly defined through dimensions such as conflict and cohesion, adaptability, organization, and the quality of communication (Lewandowski et al., 2010). The family functioning was studied in a total of eight studies (Al Ghriwati et al., 2021b; Erickson et al., 2022; Gutiérrez-Colina et al., 2017; Huang et al., 2018; Kunin-Batson et al., 2016; Moscato et al., 2025; Quast et al., 2018; Tillery et al., 2022). In the majority of studies obtained, family functioning was measured using either McMaster Family Assessment Device (Al Ghriwati et al., 2021b; Kunin-Batson et al., 2016; Moscato et al., 2025; Quast et al., 2018) or Family Environment Scale (Erickson et al., 2022; Huang et al., 2018; Tillery et al., 2022). Additionally, one study measured family functioning using the Family Adaptability and Cohesion Scale (Gutiérrez-Colina et al., 2017). These various measurement tools were also based on distinct theoretical frameworks and capture somewhat different dimensions of family environment and functioning. Therefore, it could limit direct comparability across studies and makes it more difficult to synthesize findings at the level of specific family constructs.

However, overall, better perceived family functioning, characterized by effective communication, cohesive family, fewer conflicts, was related to promotion of both physical and psychological patient outcomes. The finding is consistent with previous findings, supporting the importance of family functioning in adjustment and better HRQL (Robinson et al., 2007; Van Schoors et al., 2017; Van Schoors et al., 2019). A few studies focused on specific functions of families, instead of examining the broad concept of family functioning: parenting style, quality of parent–child relationship, and parent–child communication. Overall, poor parent–child/adolescent relationship and communication was related to poor psychological health outcomes and greater instances of substance abuse among adolescent populations.

#### 4.3.4 Identified limitations in the obtained literatures

Overall, relatively few limitations were identified across the included studies. Most studies utilized a cross-sectional design (19 of the 24), which limits the ability to determine causal relationships and directionality of relationship between family environment and patient outcomes, as well as determining the mechanisms underlying said relationship. This limitation may also stem from the predominant focus of existing studies on identifying associations between variables, rather than examining casual relationships. Future studies that examine the mechanistic pathways linking family environment to patient outcomes are needed to better establish causal relationship. Additionally, unrevealed mechanisms might originate from a lack of theoritical frameworks. In our scoping review, only six studies employed a theoretical framework, two of which are not traditional family system theories that were developed to explain the influence of family environment on patient outcomes. A future study which is based on a strong theoretical foundation is essential in filling these gaps in today’s literature. The final limitation includes reliance on self-report measurements, either proxy or self-report, and lack of objective measurements. It is reasonable to rely on self-report measures considering the characteristics of family environment variables, such as family functioning and parent–child relationship, as they are subjective; however, although this may be true, it could be beneficial to incorporate objective measures as well. Among the included studies, Murphy et al. (2016) observed maternal communication using the Iowa Family Interaction Scale (IFIRS) to measure parent-adolescent communication (Melby and Conger, 2001). Employing a third-party report who views the parent–child relationship from the sidelines may be another possibility. Only one study employed objective measures (hair cortisol) to examine the influence of family environment on survivors’ adjustment (Erickson et al., 2022). Utilizing objective measures in combination with subjective self-report measures in determining the health outcome would be recommended in the future studies, ensuring complete, accurate, and meaningful data.

#### 4.3.5 Limitations

Several limitations of this scoping review should be acknowledged. A key limitation of this review is the relatively small number of included studies (*n* = 24) in relation to the broad scope of family environment constructs examined. Although this is consistent with the purpose of a scoping review, which is to map and characterize an emerging and heterogeneous body of literature, it limits the extent to which robust conclusion can be drawn for specific sub-themes such as socio-economic status, parent–child communication, parental mental health, and family functioning. Therefore, the findings should be interpreted as indicative rather than conclusive. At the same time, the small number of eligible studies highlights an important gap in the literature and underscores the need for more focused primary research on family environment factors. Another important limitation of this scoping review is the overrepresentation of North American studies, with 16 of the 24 included studies conducted in the United States. This may reflect the extent to which the findings can be generalized to other cultural, social, and healthcare contexts. Family functioning and family environment are shaped by culturally specific factors, including the role of extend family, parent’s roles and expectations, and differences in access to care and support services (Campos and Kim, 2017; Lansford, 2022). As a result, findings derived largely from North American settings may not fully reflect family experiences in other regions. Furthermore, the included studies did not permit a more detailed consideration of variation by race and ethnicity within the North American samples. Together, these limitations highlight the need for future research including more geographically and culturally diverse populations. Finally, no conclusive evidence could be drawn regarding causal relationship between supportive family environment and health outcomes because most studies employed cross-sectional designs.

### 4.4 Implications

Given the limitations of the existing literature, it was not possible to identify a single-family environment factor that should be prioritized universally as the primary target for clinical intervention. The evidence reviewed was broad in scope but limited in depth, with considerable variation in the family environment constructs examined and no clear consensus across studies. Nevertheless, the findings consistently underscore the importance of the family environment across the illness trajectory and support the inclusion of family-focused perspectives in clinical care. In particular, regular assessment of family functioning may be especially valuable, as this was one of the most frequently examined and conceptually inclusive constructs, encompassing dimensions such as cohesion, communication, and overall family relationships. Although no single “best-fit” factor can be recommended on the basis of the current evidence, these findings suggest that attention to family functioning may help clinicians identify families who may benefit from additional support. Several family-based interventions have demonstrated promising outcomes in improving physical health by targeting health behaviors. However, a notable gap in the existing literature is the lack of clarity regarding which specific dimensions of the family environment contribute most to successful behavior change. Identifying the most influential family environment variables therefore remains an important direction for future research. Future studies should also explore a broader range of family environment constructs, examine diverse health outcomes including physical well-being, and seek to clarify causal pathways and the underlying mechanisms through which family environment influences health.

## 5 Conclusion

Family is often described as one of the most proximal and influential sources of social support for children, adolescents, and younger adults with cancer; however, its impact on health outcomes-particularly physical well-being has been underestimated and underexplored. This review contributes to existing knowledge by identifying specific family environment variables examined in prior research and emphasize the importance of supportive family functioning, favorable socioeconomic conditions, and parental psychological well-being in shaping health behaviors and outcomes that promote physical and psychological wellness in children/AYAs with cancer.
