# Supplementary material for: Family environment variables and related health outcomes in children and adolescent and younger adults affected by cancer: a scoping review
Source: Front Psychol. 2026 Jul 13;17:1843357. doi: 10.3389/fpsyg.2026.1843357 (PMC13402432; doi:10.3389/fpsyg.2026.1843357)
Supplement: Supplementary file 1 [file Table_1.DOCX]

**Table1. Literature Synthesis Table**

| Citation | Study Design | | Study Aims | Participant Characteristics | Family Environment/  Measurement | Health Outcome | Theoretical Framework | Level of Evidence |
| --- | --- | --- | --- | --- | --- | --- | --- | --- |
|  | | Non-Experimental, observational Studies | | | | | | |
| 1.Al Ghritwati, N.A., et al. 2021. (USA) | Cross-sectional, correlational design | | To examine the associations among cancer and treatment-related variables, family factors and patient HRQL after treatment completion. | 51 caregiver-CCS dyads (CCS age 18 or under, at treatment completion). | Caregiver HRQL and family functioning/The Pediatric Quality of Life-Family Impact Module (FIM), caregiver distress/Distress Thermometer | Child physical HRQL and psychosocial HRQL/PedsQL | Risk-and-resilience system | Level V |
| 2.Al Ghritwati, N.A., et al. 2021. (USA) | Cross-sectional correlational design | | To identify subtypes of family relationships and evaluate how these profiles related to child adjustment upon treatment completion | 77 CCS (age 7-14) who completed tumor directed therapy in past 7 months and caregiver | Family relationship profiles (Family closeness and discord)/NRI-RQV. | Peer relationships/PROMIS-Peer-Relationship Scale, externalizing problems/CBCL  CCS quality of life (Pediatric Quality of Life Inventory) | N/A | Level V |
| 3. Cousino et al., 2017 | Cross-sectional correlational design | | To examine illness-specific family burden as a mediator of the association between late effects of childhood cancer survivors ‘emotional and behavioral outcomes. | Childhood cancer survivors (age 10-17) two or more years off treatment and their parents | Parent-reported Impact of Family Scale (IOF), measuring the impact of the child’s health condition on the family system (financial burden, familial/social impact, personal strain, and mastery). | Behavior Assessment System for Children, second edition (BASC-2, measuring child’s externalizing and internalizing behaviors).  Youth reported PTSD, and 35-item HKI comprehensive checklist (measuring Survivors’ late effects of growth problems, reproductive problems, and vision impairments, and significant scarring) | Transactional Stress and Coping Model (Thompson & Gustafson, 1996). | Level V |
| 4. Delehaye et al., 2024 (France) | A prospective, multicenter observational (noninterventional) study, using a prospective multicenter database. | | To examine the influence of the household’s SES on school and psychological difficulties. | 1003 CCS (younger than 18 years old) | Social deprivation (household’s SES) | Psychological difficulties  (global well-being, anxiety, and depression) | N/A | Level V |
| 5. Erickson, S.J., et al. 2022. (USA) | Cross-sectional, correlational, design | | To examine the relationship between a long-term and retrospective measure of HPA activity (hair cortisol concentration) and family functioning. | 26 CCS (age 5-18) | Parent stress level/hair cortisol, Family environment/FES, Families’ social economic status | Child stress levels/hair cortisol | N/A | Level V |
| 6. Gutiérrez-Colina et al., 2016. (USA) | Cross-sectional, correlational design study using secondary data. | | To examine dyadic relationships between depressive symptoms and family functioning in families of pediatric cancer survivors. | 64 AYA cancer survivors (age 14-21), diagnosed before 18, completed treatment 2+ years previously | Caregiver perception of depressive symptoms/Depression subscale in the Brief Symptom Inventory-18, family functioning/ FACS | AYA depressive symptoms/ the Behavior Assessment System for Chiildren-2^nd^ Edition Self-Report. | N/A | Level V |
| 7. Hocking et al., 2017. (USA) | Cross-sectional, mixed method design study | | To obtain caregivers perspectives on survivor social competence and identify potential risk and resistance factors. | Caregivers of pediatric brain tumor survivors (age 8-17) | Family functioning/McMaster Family Assessment Device (FAD-GFS), parental interpretation of behavior and parenting style(interviews). | CCS executive functioning/The Global Executive Composite from the Behavior Rating Inventory of Execute Function, survivor social adjustment (PedsQL4.0) | Model of social competence in children w/ brain disorder. | Level V |
| 8. Huang, J. et al., 2018. (USA) | Cross-sectional, correlational design | | To investigate the influence of family on children’s health status, symptoms and family strain in survivors of childhood acute lymphoblastic leukemia (ALL). | 213 CCS (age 8-23) and parents | Family cohesion/Family Environment Scale, parental emotional distress/The Brief Symptom Inventory, parental protective behavior/the Parent Protection Scale,  Maternal education | Child HRQOL/the proxy version of the PedsQL-Generic Core Module version-4  Symptom burden/ the proxy-version of the PedsQL-Cancer Module version (perceived abnormal physical, emotional, and cognitive symptoms) | N/A | Level V |
| 9. Kim & Im, 2015. (South Korea) | Cross-sectional correlational study | | To examine the psychosocial problems in childhood cancer survivors in Korea and their connection to family management style | 158 CCS (age 4-16) and parents | Family management style/ Family Management Measure | Psychosocial problems/the Pediatric Symptom Checklist | Family Management Style Framework | Level V |
| 10. Kim et al., 2020. (USA) | Exploratory, mixed method designs study | | To examine associations among sleep problems in children with cancer, parental behavior, and sleep hygiene throughout the cancer treatment, and evaluate aspects of children’s sleep associated with sleep problems during and after cancer treatment. | 80 parents of children with cancer (age2-10, in active treatment) | Parental accommodation behaviors in sleeping, psychological factors (anxiety) | Children’s sleep disturbance, improvement of children’s sleep | N/A | Level V |
| 11. Kunin-Baston. et al., 2016. (USA) | Prospective, longitudinal study | | To examine the prevalence and predictors of anxiety and depression after the completion of treatment among childhood cancer survivors of acute lymphoblastic leukemia. | 160 children (age 2-9) w/ standard risk ALL and their parents | Family language (Spanish or not),  Family functioning/ FAD-GFS  Parental Coping/Coping Health Inventory for Parents (CHIP). | Anxiety and depression symptoms after treatment completion/ the Behavioral Assessment System for Children, 2^nd^ Edition: Parent Report Scale. | N/A | Level III |
| 12. Moscato et al., 2025. (USA) | Cross-sectional, secondary analysis | | To evaluate how family functioning is associated with QOL among children with differing tumor types and treatment intensities soon after treatment completion. | Youth aged 8-14 years who completed tumor-directed treatment within six months prior to enrollment for a BT or a non-CNS solid tumor and their caregivers. | Family functioning (FAD-GFS) | Physical and psychosocial QOL/Pediatric Quality of Life Inventory (PedsQL), 4.0. | N/A | Level V |
| 13. Murphy et al., 2016. (USA) | Longitudinal, correlational design | | To examine adolescent and maternal post-traumatic stress symptoms and maternal communication from time near cancer diagnosis to 12 months follow-up to identify potential risk factors for adolescent PTSS. | 41 Adolescents w/ cancer (age 10-17) and their mothers. | Maternal PTSS/ the Impact of Events Scale-Revised,  Observed maternal communication (Harsh and withdrawn and solicits/ validations)/ the Iowa Family Interaction Scale | Adolescent PTSS/ the Impact of Events Scale-Revised. | N/A | Level III |
| 14. Patterson, V. et al., 2024. (USA) | Longitudinal, correlational design study. | | To examine associations between early sociodemographic, family stress, and general mother-adolescent communication on the HRQOL of survivors at 5 years post diagnosis. | 50 children (age 5-17), 80 mothers | Sociodemographic score, mother stress/ Perceived Stress Scale,  Mother-adolescent communication/ PACS | Survivor HRQOL/PedsQL 4.0. | N/A | Level III |
| 15. Quast et al., 2018. (USA) | Prospective longitudinal observational study | | To examine associations between family functioning at the end of tumor-directed treatment and HRQL of pediatric brain tumor survivors approximately 9 months later | 35 pediatric brain tumor survivors and mothers | Survivor reports of Family functioning/FAD-GFS  Parents perception of family’s ability to manage a child’s condition in daily life/ Family management Measure  Parents perception of challenges in manage illness-related demands. /Family life difficulty (FLD) subscale | Survivor HRQL, both survivors and mother rated, / PedsQL 4.0. | N/A | Level III |
| 16. Racine et al., 2018. (Canada) | Cross-sectional observational study | | To examine the association between psychosocial family risk factor, parental psychological distress, and health-related quality of life in pediatric cancer survivors. | 52 CCS (mean age 11.92) and parents | Parent psychological distress/The Brief Symptom Inventory, psychosocial family risk/Psychosocial Assessment Tool | Child HRQOL, both parent and child reported/ PedsQL 4.0 | N/A | Level V |
| 17. Sim et al., 2024. (USA) | Cross-sectional, correlational design study | | To examine how multilevel social factors affect patient-reported outcomes in children under 18 who survived cancer. | 293 CCS and caregivers | Family dynamics(conflicts)/ Family Relationship Index of the Family Environment Scale,  Parent depression, stress, fatigue, sleep issues, mobility, and sleep problems/PROMIS  Residence in poverty area/socioeconomic status, physical environment via the Country Health Ranking & Roadmaps) | CCS depression, stress, fatigue, sleep issues, mobility, and sleep problems/ PROMIS-Pediatric Short Form. | N/A | Level V |
| 18. Tillery, R et al., 2020. (USA) | Observational cross-sectional design | | To identify patterns of parent-child relationship characteristics between young survivors of childhood cancer and their caregivers. | 165 youth (age 10-18) w/ history of cancer and caregivers | Patterns of parent-child relationship functioning (involvement, Attachment, Communication, Parenting confidence, and relational Frustration)/ Parenting Relationship Questionnaire | PTSS levels/ UCLA PTSD Reaction Index for DSM-IV, internalizing symptoms/ The Behavior Assessment System for Children, 2^nd^ Edition, social functioning. | N/A | Level V |
| 19. Tilley, R. et al., 2022. (USA) | Longitudinal, correlational design | | To examine substance use among AYA survivors of pediatric cancer and AYA without a history of chronic or life-threatening illnesses, and to explore links between demographic, medial, caregiver-AYA, and family-system factors with AYA substance use patterns. | 171 AYA survivor-caregiver dyads, 118 comparison caregiver dyads. (Child ages 12-23) | Caregiver-AYA relationship Quality (attachment, communication, involvement, parenting confidence, and relational frustration/ Parenting Relationship Questionnaire. | Polysubstance abuse (Risky drinking and smoking)/Practical Health Behavior Measure | N/A | Level III |
| 20. Van Laere et al., 2023.  (Belgium) | Three-wave, longitudinal, correlational design study | | To examine the directionality of effects among parental sense of incompetence, parenting dimensions, and survivor psychological functioning. | 125 CCS (age 14-24), 114 mothers/91 fathers. | Parental incompetence/12-item Sense of Competence Scale of the Parenting Stress Index Questionnaire, Parenting dimensions (responsiveness, psychological control/, Child Report of Parent Behavior Inventory and over protection/ both parents’ and survivors’ self-report using seven-items of the Multidimensional Overprotective Parenting Scale and one item of the Egna Minnen Betraffande Uppofostran. (Self-reporting questionnaire assessing parenting) | CCS depressive symptoms/ 2-item version of the Center for Epidemiologic Studies Depression Scal, fear of cancer recurrence,  Benefit Findings/ Benefit Scale of Benefit and Burden Scale for Children. | Belsky’s process model of parents | Level III |
| 21. Winning et al., 2022. (USA) | Observational, cross-sectional study | | To examine if maternal parenting behaviors influence the relationship between CNS direct treatment and adjustment among pediatric cancer survivors at 3 years post-diagnosis or relapse. | 84 CCS (age 5-17) receiving CNS direct treatment and their mothers | Children report on maternal parenting behaviors (Maternal warmth, behavioral and psychological control)/ Parnting Behavior Inventory (CRPBI-30) | Externalizing/internalizing problems, academic competence, social competence/ Child Behavior Checklist, | N/A | Level V |
| 22. Zakovic et al., 2024. (Switzerland) | Cross-sectional correlational design study | | To assess the prevalence of parental smoking among CCS and investigate its association with respiratory outcomes. | Parents of CCS (age 16 or younger) who survived 5+ years after diagnosis | Parental smoking | Recurrent UTRI, lower respiratory symptoms (chronic cough, wheeze during exercise, etc) | N/A | Level V |
| 23. Zheng et al., 2022. (China) | Cross-sectional design Study. | | To examine multiple health behaviors among Chinese CCS and explore the individual, interpersonal, and home environmental factors affecting health behaviors. | 204 CCS (age 4-18) | Family support/ Chinese version of Perceived Social Support, Residential location, household income, father/mother education level, parents’ marital status | CCS’s health behaviors including smoking, drinking, drug use screen time, internet addiction, dietary behaviors that may lead to overweight or obesity, (using the Chinese Adolescent-related Health Surveillance Questionnaire), physical activity (using the Children’s Leisure Time Activities Study Survey-Cheese), sleep behaviors using the Pittsburgh Sleep Quality Index. | Social ecological model. | Level V |
|  | | Experimental Design Study | | | | | | |
| 24. Bidstrup, B.E. et al., 2023. (Denmark) | Randomized Control Design | | (1) to examine the effect of FAMOS intervention on trauma-related behavior in the sub-group of children who finished cancer treatment. (2) to examine whether reduced depressive symptoms in parents mediated the effect of FAMOS on the trauma-related behaviors. | Families of children aged 0-6 years old who had ended curative intended treatment within 4 months: 51 families in intervention, 58 in control group. | Mother depressive symptoms | Trauma related behavior |  | Level I |

*CCS refers childhood cancer survivors, ** FAD-GFS: the McMaster Family Assessment Device- General Functioning Scale, PedsQL: Pediatric Quality of Life Inventory, FES: Family Environment Scale, FACS: Family Adaptability and Cohesion Scale, NRI-RQV: Network of Relationship Inventory-Relationship Quality Version.

Table2. Summary of Findings

| **Family Environment -Physical** | **Family Environment-Psychosocial** | **Physical Health Outcomes** | **Psychological Health Outcomes** |
| --- | --- | --- | --- |
| Mothers’ education level, insurance status, household income, and residential location (Zip code, rural vs Urban). Marital status, Parental sleep strategies (behaviors that parents use to put their children to sleep), parental smoking, | Family Dynamic (family conflict), Caregivers’ anxiety, depression, and fatigue. Parental sense of incompetence, Parenting (responsiveness, psychological control, and overprotection), Family functioning, Family support, Maternal PTSS, maternal communication (harsh/withdrawn, family burden, Parent-child relationship. | CCS’s health behaviors (smoking, drinking, drug use, screen time, internet addiction, dietary behaviors that may lead to overweight or obesity), sleep disturbance, recurrent UTRI (either recurrent sinusitis, OM or both) and lower respiratory symptoms (either chronic cough, at least one episode of wheeze in the past 12 months, or wheeze during exercise), neurocognition outcomes among brain tumor survivors. | CCS’ depression, psychological stress, fatigue, sleep disturbance, positive affect, and mobility domains, Survivors’ psychological functioning (depressive symptoms, fear of cancer recurrence, and benefit finding). Symptom burden, Survivors’ social adjustment, survivors’ executive functioning, psychological well-being, PTSS, Internal and externalizing symptoms, Physical and psychosocial QOL. |
